# A case of confirmed mosaic tuberous sclerosis complex with isolated shagreen patch as the sole cutaneous manifestation

**DOI:** 10.1016/j.jdcr.2025.10.032

**Published:** 2025-10-28

**Authors:** Elyse Pitock, Amanda Doyle

**Affiliations:** a Russak Dermatology, New York, New York; b Department of Dermatology, The Mount Sinai Hospital, New York, New York

**Keywords:** mosaic tuberous sclerosis, shagreen patch, tuberous sclerosis complex

## Introduction

Tuberous sclerosis complex (TSC) is an autosomal dominant genetic disease that causes hamartomas to grow in the brain, eyes, kidneys, lungs, skin, and heart, as well as some cancerous renal tumors.^1^ Presentation is highly variable between patients, but common symptoms include epilepsy and intellectual disability. Cutaneous findings can include hypomelanotic macules, facial angiofibromas and fibrous cephalic plaques, shagreen patches, ungual fibromas, and gingival fibromas with patients presenting with many of these cutaneous findings.^2^ These skin abnormalities can be the first indicator for some patients with TSC and are observed in a high percentage of individuals with this condition. In fact, at diagnosis, 58.1% of patients present with cutaneous findings (with the most common lesion being hypomelanotic macules in 92% of those).^3^ In those with few or no cutaneous or neurological signs and symptoms, diagnosis may be significantly delayed and/or the condition may go undiagnosed entirely. In TSC patients, the primary concerns are cutaneous, neurological, cardiac, renal, and pulmonary.^4^ We present a unique case where there was a delayed diagnosis of TSC with minimal cutaneous findings and no neurological symptoms in a young, asymptomatic, otherwise healthy woman.

## Case report

**Fig 1:**
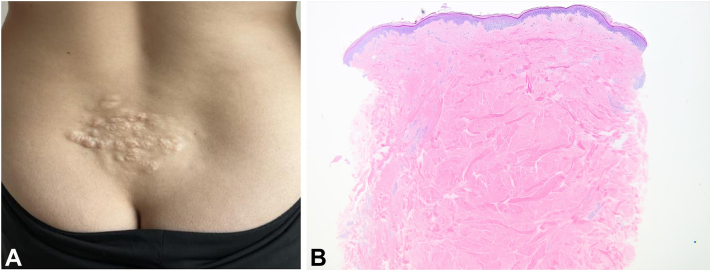
**A,** Shagreen patch on patient's lower back. **B,** Histopathology of shagreen patch on patient’s lower back found during skin cancer check.

During a routine skin cancer check, a healthy 28-year-old woman was found to have a collagenoma on her lower back with no other cutaneous findings (Fig 1, *A* and *B*). Patient reported the collagenoma developed during childhood. At the time of the examination, the lesion appeared clinically consistent with a shagreen patch, raising concern for the possibility of tuberous sclerosis. The patient reported no symptoms suggestive of internal involvement. Given its isolated finding, it was a highly unusual case, and histological confirmation was necessary before moving forward with genetic screening/further evaluation. Histology confirmed that the lesion was a collagenoma, specifically a shagreen patch if the patient did have tuberous sclerosis. Initial standard blood-based targeted gene panels for TSC1 and TSC2 were negative at age 28. A brain magnetic resonance imaging showed subtle nonenhancing foci with abnormal fluid attenuated inversion recovery signal involving the right posterior parietal cortex and along the left parieto-occipital sulcus. These findings were consistent with cortical tubers. A kidney ultrasound showed a 1.6 cm × 1.4 cm nonspecific soft tissue attenuation on her left kidney. A subsequent magnetic resonance imaging identified a mass suspicious for a renal cell carcinoma, most likely a papillary renal cell carcinoma. A computed tomography scan of the lung showed findings of possible multifocal micronodular pneumocyte hyperplasia. Pulmonary function testing was normal.

A biopsy of the kidney mass was attempted but was aborted due to interposition of bowel.

Physicians recommended partial left nephrectomy. After surgery, pathology showed a lipid-poor, spindle-cell-predominant angiomyolipoma. Cortical tumors, angiomyolipoma, and shagreen patch confirmed a clinical diagnosis of tuberous sclerosis. MSK-IMPACT (Integrated Mutation Profiling of Actionable Cancer Targets) testing was performed. No germline mutation was identified, but the 2 angiomyolipomas shared a deep intronic pathogenic variant (present in blood at <5% allele frequency), and she was determined to have a mosaic TSC. The patient continues to follow up with dermatology, renal, and pulmonary specialists and is doing well.

## Discussion

The clinical diagnostic criteria for TSC are 2 major features and 1 minor feature (Table I). For patients not diagnosed in utero, pursuit of a diagnosis is usually initiated by the discovery of neurological and/or cutaneous features.^4^ Up to 97% of TSC patients have hypomelanotic macules. Less ubiquitous but still common are facial angiofibromas (74.5%) and shagreen patches (48%).^2^ Each of these features, along with ungual fibromas, are major features of TSC. The presence of 1 feature can indicate the presence of other major/minor features and may warrant referral to genetics and/or neurology.^2^ TSC is associated with several pulmonary manifestations, including lymphangioleiomyomatosis, multifocal micronodular pneumocyte hyperplasia, and chylous effusions. Thus, patients should be evaluated for pulmonary involvement.^5^

**Table I:** Diagnostic criteria for tuberous sclerosis complex

Major features	Minor features
Hypomelanotic macules (≥3; at least 5 mm diameter)	“Confetti” skin lesions
Angiofibroma (≥3) or fibrous cephalic plaque	Dental enamel pits (≥3)
Ungual fibromas (≥2)	Intraoral fibromas (≥2)
Shagreen patch	Retinal achromic patch
Multiple retinal hamartomas	Multiple renal cysts
Multiple cortical tubers and/or radial migration lines	Nonrenal hamartomas
Subependymal nodule (≥2)	Sclerotic bone lesions
Subependymal giant cell astrocytoma	
Cardiac rhabdomyoma	
LAM∗	
Angiomyolipomas (≥2)∗	

Definite TSC: 2 major features or 1 major feature with 2 minor features.

Possible TSC: Either 1 major feature or ≥2 minor features.

*LAM*, Lymphangiomyomatosis.

∗ A combination of the 2 major clinical features, LAM and angiomyolipomas, without other features does not meet criteria for a definite diagnosis.

While unusual, between 10 and 15% of TSC patients do not have germline mutations.^6^ In these mosaic cases, blood tests will return false negatives.^7^ Accurate diagnosis of mosaic TSC may require next-generation genetic testing of actual neoplasms. Accurate diagnosis is also important for potential heritability. A parent with a germline TSC mutation has a 50% risk of offspring transmission. However, the risk of transmission in mosaic TSC appears to be less frequent, particularly with lower levels of mosaicism, and occurs at a frequency of 1 of 12 live births (8%), according to a recent study evaluating a mosaic TSC cohort.^8^ This case underscores that even in the presence of negative genetic testing and isolated cutaneous findings suggestive of TSC, serious internal manifestations are possible. Physicians who identify a major feature with a negative blood test in a patient with possible mosaic TSC should inquire about relevant neurologic, renal, ophthalmologic, dermatologic, and pulmonary symptoms. This conversation should include education around the risks and benefits of a comprehensive workup for TSC. If patients choose to undergo further evaluation, an assessment to rule out systemic features, including appropriate imaging, may include neurologists, nephrologists, dermatologists, pulmonologists, ophthalmologists, and/or genetic counselors.

This is an example of a more subtle and unique presentation of a more common genodermatosis. We present this case to help raise awareness and increase suspicion for more atypical presentations of TSC.

## Conflicts of interest

Dr Doyle is a consultant for Merz, SkinCeuticals, Skinbetter, Allergan, and Vaseline. Ms Pitock has no conflicts of interest to declare.
